# Triple Jeopardy: Rapidly Progressive Glomerulonephritis Induced by Triple Seropositive Disease—A Rare Case

**DOI:** 10.1155/2022/2032525

**Published:** 2022-11-14

**Authors:** Apurva Vedire, Gautham Upadrasta, Ndausung Udongwo, Faseeha Rehman, Mohammad A. Hossain

**Affiliations:** Department of Medicine, Jersey Shore University Medical Center, Neptune City, NJ 077053, USA

## Abstract

The double-positive disease is the co-occurrence of antiglomerular basement membrane (anti-GBM) disease and antineutrophil cytoplasmic antibodies (ANCAs) and is an uncommon cause of renal failure. Our case of triple-positive disease is an even rarer cause of isolated renal failure, as it includes anti-GBM, antimyeloperoxidase (MPO), and antiproteinase 3 (PR3). We present a case of a 62-year-old Caucasian male with a history of multiple comorbidities, who presented to the emergency department (ED) with worsening dyspnea on exertion that started about one month prior to admission. He was found to be in renal failure secondary to triple-positive disease. We believe that the likely mechanism of our patient's triple-positive disease was a drug-induced ANCA vasculitis overlapping with Goodpasture's syndrome. We believe our case to be a valuable addition to the literature as it is a rare overlap syndrome without a previously established disease course or etiology.

## 1. Introduction

Rapidly progressive glomerulonephritis resulting from the coexistence of antiglomerular basement membrane (anti-GBM) disease and antineutrophil cytoplasmic antibodies (ANCAs) is an unusual cause of renal failure and is regarded as a “double-positive” disease (DP) [1]. DPD is a rare disease that was first coined in the 1980s by Omotoso et al. [2]. About 20–40% of anti-GBM diseases are equally ANCA positive and 5–14% vice versa [3, 4]. A “triple positive” disease (anti-GBM, myeloperoxidase (MPO), and proteinase 3 (PR3)) causing an isolated renal failure is even rarer [3]. Due to its rarity, its natural history, prognosis, and appropriate management remain unknown [3]. We present a case of a 62-year-old Caucasian male who presented to the emergency department with dyspnea on exertion for about a month. Serology and renal biopsy-proven tests confirmed a triple-positive disease.

## 2. Case Presentation

A 62-year-old Caucasian male with a past medical history of hypothyroidism, vitamin D deficiency, hyperlipidemia, prediabetes, coronary artery disease status postright coronary artery stent placement, and subarachnoid hematoma presented to the emergency department (ED) with worsening dyspnea on exertion that started about one month prior to admission. Symptoms started a few days after flu-like symptoms. It was associated with persistent productive cough, fatigue, nausea, vomiting, diarrhea, generalized weakness, decreased appetite, change in the color of his urine, and myalgia. He denied any chest pain, dizziness, headache, lower extremity edema, recent travel history, or sick contacts. He worked as a supervisor in a construction company. He drank about 1-2 bottles of beer daily. He smoked 2 packs of tobacco per day (60 pack-years; discontinued in 2020). He denied any use of illicit drugs. Family history was remarkable for gastric carcinoma in the mother. He received 2 doses of the severe acute respiratory syndrome coronavirus 2 (SARS-CoV-2) vaccine. Of note, he endorsed taking daily high doses (unspecified) of ibuprofen and naproxen about 3–4 times a day for 30 days, due to persistent severe myalgia. His other home medications included alprazolam, atorvastatin, oxycodone-acetaminophen, levothyroxine, aspirin, and cyclobenzaprine.

Vitals were blood pressure, 157/64 mmHg; heart rate, 94 beats per minute; respiratory rate, 16 breaths per minute; oxygen saturation, 96% on ambient air. Body mass index (BMI) was 25.54 kg/m^2^. The cardiopulmonary examination was unremarkable except for tachycardia. There were no murmurs, rubs, or gallops. Lung sounds were audible on auscultation. There was no jugular venous distention or lower extremity edema. Bilateral involuntary rhythmic tremors in his hands at rest and with the movement were noted. Laboratory studies (Table 1) revealed low hemoglobin, mild leukocytosis, highly elevated blood urea nitrogen (BUN), serum creatinine, and hypoalbuminemia. Cr level was 0.95 mg/dL, within normal limits 5 months prior.

**Figure 1:**
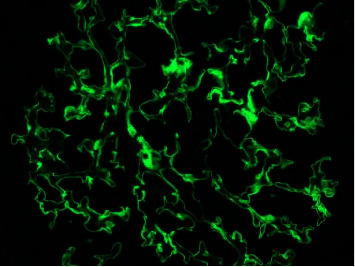
Immunofluorescent antibody staining of glomeruli demonstrating linear deposition of IgG anti-GBM antibodies.

**Figure 2:**
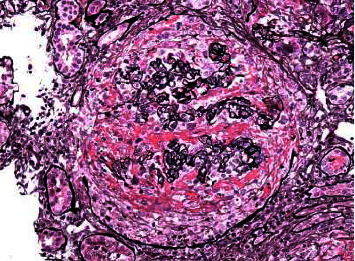
Light microscopy of glomeruli demonstrating the crescentic pattern of infiltration.

The electrocardiogram and chest X-ray was unremarkable. Computed tomography of the chest and abdomen showed nonobstructing right renal calculi with bilateral perinephric stranding, confirmed with renal ultrasound. There was no hydronephrosis. The patient had been oliguric and was admitted for the management of acute kidney injury (AKI). Within 12 hours of the presentation, he was placed on a bicarbonate drip and then underwent urgent hemodialysis (HD). 16 hours later, he had a fever of 102°F with leukocytosis and was started on empiric antibiotics. Blood culture grew Enterococcus faecium only in 1 bottle. Despite undergoing 3 rounds of scheduled HD sessions (Tuesday, Thursday, and Saturday), there was no improvement in his Cr level or urine output, prompting further workup. Autoimmune panel results are shown in the following (Table 2):

A diagnosis of double-positiveanti-GBM and ANCA was made. There was a collective management approach involving nephrology, infectious disease, and hematology. The patient was started on plasmapheresis in addition to steroids, with recommendations to obtain a renal biopsy to help determine the duration of therapy (7 days or 2–3 weeks). On day 6, a right renal biopsy was obtained, and the result confirmed severe anti-GBM (Goodpasture syndrome) with 100% glomerular involvement (Figures 1 and 2). Cyclophosphamide was added to his medication regimen. His hospital course was complicated with new-onset type 2 diabetes and hyperkalemia, which were both managed appropriately. His renal function improved and stabilized. He was discharged in a stable condition after placement of Permacath with plans to continue outpatient plasmapheresis (14 sessions), cyclophosphamide, and dialysis. Two weeks later, he returned to the ED with complaints of diarrhea and was diagnosed with Clostridium difficile colitis. He was discharged on oral vancomycin and has remained stable on outpatient follow-up visits.

## 3. Discussion

Double antibody-positive disease (DAPD), exhibiting antiglomerular basement (anti-GBM) and antineutrophil cytoplasmic antibodies (ANCAs) are auto-antibody mediated small vessel vasculitis with ideal preference to renal and pulmonary systems [5]. Several factors such as smoking, viral infection, or hydrocarbon compounds could precipitate this disease [5, 6]. It is a variant of Goodpasture syndrome (GPS). Anti-GBM disease is characterized by an autoimmune response against the *α*3 chain of type IV collagen on the GBM in GPS [7]. Contrastingly, myeloperoxidase (MPO) and proteinase 3 (PR3) antibodies are features of ANCA-associated vasculitis (granulomatosis with polyangiitis, microscopic polyangiitis, and eosinophilic granulomatosis with polyangiitis) [5]. ANCA-associated vasculitis (AAV) can include the following features: purpura (granulomatous and nongranulomatous skin lesions), neuropathy, acute renal failure (due to neutrophil infiltration into renal tissue), hemoptysis (due to alveolar hemorrhage and nasopharyngeal/oropharyngeal tract hemorrhage), as well as more nonspecific features, such as arthralgias and fatigue. Rapidly progressive glomerulonephritis with or without pulmonary involvement is an independent feature of either anti-GBM or ANCAs, making DAPD patients at risk of worsening renal outcomes [5, 7, 8].

DAPD has a reported incidence of 0.00006% worldwide and accounts for about 25% of cases of GPS [8, 9]. In addition, an estimated 20–40% of GPS patients have a coexistence of ANCAs [1, 4]. It is more common in Caucasians and in ages between 50–59 and ≥70 years, with a median age of 57.1 years [4, 8, 10]. Patients with DAPD have a more severe rapidly progressive glomerulonephritis when compared to those with isolated anti-GBM [4, 8]. Although, the former shows better renal prognosis & long-term survival with an increased rate of relapse [8, 9, 11]. Similar to patients with GPS, they exhibit early morbidity and mortality [11]. In a recent systematic review published by Philip et al. in 2021, assessing 538 DAPD patients using 90 articles, 72.1%, and 20.5% were positive for MPO and PR3, respectively. Only 2.9% of these patients were triple-positive (anti-GBM + ANCAs (MPO + PR3)) [8]. Furthermore, McAdoo et al. in a large retrospective cohort study suggested that anti-GBM disease was the dominant early form of DAPD [11].

The pathophysiology of this disease has been speculated because of an initial injury to the BM triggered by ANCAs, exposing the antigens (NC1 domain of *α*3 chain of type IV collagen) to the development of antibodies against anti-GBM (GPS) [7]. Also, cross-reactive T cell responses on the BMs triggered by both antibodies (anti-GBM/ANCAs) and the release of MPO during apoptosis by neutrophil extracellular traps (NETosis) in isolated anti-GBM disease have also been proposed [8, 12, 13]. A triple-positive mechanism is still unclear.

Diagnostic criteria for GPS include 1: the presence of clinical symptoms, 2: positivity serology tests, and 3: histological findings from the kidney or lung biopsy. These findings include linear staining with IgG under immunofluorescence, using light microscopy [14]. In addition, sclerotic glomeruli are present in DAPD likely due to the coexistence of ANCAs (MPO-ANCA) [8]. Intrarenal arteritis has also been described in PR3-ANCA double-positive patients [1].

DAPD is one of the 10 indications for therapeutic plasmapheresis (TP). A combination of this treatment with immunosuppressive therapy like steroids and cyclophosphamide has shown good benefits in several reported retrospective studies and case reports [9, 15]. The duration of this therapy is also based on disease severity, with longer durations (2–3 weeks) aimed at patients with DAPD presenting severe clinical symptoms. Although good clinical outcomes with combination therapy versus steroids alone have been reported, some authors still believe that DAPD patients respond poorly to this therapy and have poor renal end results [15, 16].

Our patient is a Caucasian male who presented to the ED with complaints of dyspnea on exertion a few days after flu-like symptoms. We initially speculated that his acute kidney injury was a result of his use of high doses of ibuprofen & naproxen use plus the rhabdomyolysis as was evident with elevated creatinine kinase level. Renal failure was refractory to maintenance fluid and continuous renal replacement therapy, prompting further workup. Serology tests with renal biopsy-proven results confirmed a “triple positive” (anti-GBM + MPO/p-ANCA + PR3/c-ANCA) GBM disease. Only about 2.9% of this group of patients have been reported [8].

A possible hypothesis is that this was the rare co-occurrence of drug-induced ANCA vasculitis and Goodpasture syndrome in the same patient. Drug-inducedANCA-associated vasculitis (AAV) does not yet have a well-established pathogenesis [17], so it is fair to say that we do not yet know which drugs could be capable of triggering the involved mechanisms. The role of NETs or neutrophil extracellular traps has been described in drug-induced AAV. Infectious factors and proinflammatory cytokines such as TNF are involved in the stimulation of neutrophils to form NETs, which are regulated through degradation by serum endonuclease DNase I. DNase I activity was seen to be reduced in drug-induced AAV, and this dysregulation of NETs can lead to ANCA production. Some of the commonly implicated drugs are cocaine with levimasole, hydralazine, biological agents, and antithyroid drugs (ATDs). Atorvastatin has also been associated in some cases, with a medication that our patient was also seen to be taking [17]. We found the case of a 60-year-old man, in whom MPO ANCA positivity was attributed to statin use [18]. Another case of a 54-year-old man with ANCA-positive vasculitis, positive for ANA and anti-MPO after simvastatin/ezetimibe [19]. Also, the case of a 45-year-old man with MPO ANCA positive vasculitis after atorvastatin [20]. MPO positivity was seen in a case of phenytoin-associated vasculitis, which was treated successfully with steroids and cyclophosphamide [21]. Steroids and cyclophosphamide were a part of our patient's treatment regimen as well.

Dual ANCA is extremely rare outside the setting of drug-induced ANCA and is in fact, only seen every now and then, even due to drugs [22]. Such cases have been seen in hydralazine use [23] and propylthiouracil use [24]. Our case was unique in that both MPO and PR3 positivity was seen together in the setting of statin use, along with the simultaneous presence of Goodpasture syndrome as well. As a result of this association in the literature between atorvastatin and AAV, the patient's atorvastatin was discontinued, and the patient is being followed by nephrology and his PCP. Currently, the patient remains dialysis dependent.

There are no established markers in either laboratory or clinical findings to differentiate primary AAV from drug-induced AAV with certainty. However, some retrospective studies comparing ATD-induced AAV (arguably the most common drug-induced AAV) and primary AAV did reveal certain patterns. Serum-positive ANA was seen more frequently in ATD-induced AAV, as well as a tendency to have multiple target antigens including MPO and PR3. In primary AAV, ANCAs were generally recognized as either MPO or PR3 [17]. Such findings support our hypothesis that this was a case of drug-induced AAV as opposed to a primary AAV.

Our patient as mentioned of course, also had a significant increase in CK levels >4000 on presentation. It is also of interest to note a seemingly insignificant, however, possibly relevant detail in the presentation of our patient, where he stated that his symptoms all began after a flu-like illness. A history of upper respiratory infections has been noted in other patients with ANCA-associated glomerulonephritis as well. The mechanism suggested explaining this observation in individuals with a genetic predisposition and infections that could trigger NET production and MPO exposure [25].

Thus, we believe that the etiology of our patient's triple positive condition was likely multifactorial, with statin use as the culminating factor in the cascade of contributing events. This case also emphasizes the importance of keeping in mind a broad array of contributing factors while taking patient history. Only through a thorough review of each unique patient presentation can we uncover new patterns that could then be studied to help us in prevention.

## 4. Conclusion

Multiple mechanisms have been hypothesized when it comes to drug-induced AAV, but it is certain that all the mechanisms involved are not yet known. The importance of unearthing more cases of drug-induced AAV becomes even more significant in such a scenario where studying new drugs causing these cases could lead to the discovery of novel mechanisms that have yet not occurred to us. Additionally, the matter of what predisposed this individual to the development of such an autoimmune condition is certainly a matter of interest, especially given that Goodpasture syndrome has been notorious for developing with an abrupt onset and no prior indicators. If we were able to unearth certain patterns in the presentation of these patients, perhaps we could be more vigilant in identifying patients at risk for both Goodpasture syndrome as well as AAV. In cases such as these, where timely treatment is of the essence, such a discovery would indeed make a true difference in the disease course of these patients.

## Figures and Tables

**Table 1:** Initial laboratory results.

Test name	Result	Reference value
Hemoglobin	10.2 g/dL	13.2–17.5 g/dL
White blood cell (WBC) count	12.2 × 10^3^/uL	4.5–11 × 10^3^/uL
BUN	122 mg/dL	5–25 mg/dL
Creatinine	9.14 mg/dL	0.61–1.24 mg/dL
Sodium	130 mmol/L	135–146 mmol/L
Anion gap	17 mmol/L	5–13 mmol/L
Bicarbonate	14 mmol/L	24–31 mmol/L
Phosphorus	7.6 mg/dL	2.5–4.6 mg/dL
Total protein	5.5 g/dL	6.0–8.0 g/dL
Albumin	2.1 g/dL	3.5–5.0 g/dL
Total creatine kinase	4,720 iU/L	22–232 iU/L
Venous blood gas pH	7.279	7.320–7.420
Urinalysis: glucose	50 mg/dL	Negative
Blood	Large	Negative
Protein	100 mg/dL	Negative
WBC	Too numerous to count	0–2/HPF
Red blood cell	Too numerous to count	0–2/HPF
Leukocyte esterase	Moderate	Negative
Bacteria	Many	None

**Table 2:** Results of autoimmune workup.

Test name	Result	Reference value
Antiglomerular basement membrane (GBM) antibody	Positive: 3.5 AI	<1.0 AI
Antinuclear antibody (ANA)	Positive	Negative
ANA titer	1 : 320	<1 : 40
Antineutrophil cytoplasmic antibody (ANCA)	Positive	Negative
Myeloperoxidase antibody (MPO)	Positive: 4.6 AI	<1 AI
Proteinase-3 antibody (PR-3)	Positive 4.3 AI	<1.0 AI
Hepatitis panel	Negative	Negative
Immunofluorescence	No monoclonal spike	No monoclonal spike

## Data Availability

The data that support the findings of this study are available from the corresponding author upon reasonable request.
